# Microbiota awareness and fermented food consumption among university students: evidence of a knowledge-behavior gap

**DOI:** 10.1038/s41598-026-55444-6

**Published:** 2026-05-28

**Authors:** Ayşe Nur Kahve, Hande Yazıcıoğlu Çalışan

**Affiliations:** Department of Sports and Health, Aksaray University, Aksaray, Turkey

**Keywords:** Gut microbiota, Fermented foods, Feeding behavior, Nutrition education, Students, Diseases, Gastroenterology, Health care, Microbiology

## Abstract

Fermented foods are traditional functional foods that may beneficially influence gut microbiota composition and contribute to overall health. However, it remains unclear whether microbiota-related awareness is associated with regular consumption of fermented foods. This study aimed to examine the association between microbiota awareness and fermented food consumption among university students. A cross-sectional study was conducted among students enrolled in sports science programmes. Data were collected using a sociodemographic questionnaire, the Microbiota Awareness Scale, and the Fermented Food Consumption Index. Overall microbiota awareness levels were relatively high, particularly in domains related to general microbiota knowledge and probiotic–prebiotic concepts. Despite this, fermented food consumption was generally low among participants. A weak but statistically significant negative correlation was observed between total microbiota awareness scores and fermented food consumption (Spearman’s r = − 0.28, *p* < 0.01). In contrast, strong positive correlations were found between microbiota awareness and both general microbiota knowledge (r = 0.85, *p* < 0.001) and probiotic–prebiotic knowledge (r = 0.82, *p* < 0.001). Students reporting gastrointestinal complaints had significantly higher awareness scores than those without such symptoms (*p* < 0.05). These findings indicate a discrepancy between microbiota-related knowledge and actual dietary behaviour, consistent with the intention–action gap described in behavioural nutrition literature.

## Introduction

For many years, human health was primarily examined through individual physiological systems. However, increasing scientific interest in the gut microbiota has substantially changed this perspective. The gut microbiota, which consists of trillions of microorganisms inhabiting the gastrointestinal tract, plays an active role in numerous physiological processes. These include immune regulation, metabolic balance, control of inflammatory responses, and modulation of neurological pathways^1,2^. Accordingly, the microbiota is now considered a key component of a holistic understanding of health rather than merely a digestive element.

The composition and diversity of the gut microbiota are influenced not only by genetic factors but also by everyday lifestyle behaviors. Among these, dietary habits represent one of the most powerful and modifiable determinants. Diets rich in dietary fiber, together with regular consumption of fermented and probiotic foods, have been associated with increased beneficial microorganisms and greater microbial diversity^3^. In contrast, frequent intake of highly processed foods and imbalanced dietary patterns has been linked to adverse alterations in the gut microbiota, commonly described as dysbiosis.

Probiotics are defined as ‘live microorganisms that, when administered in adequate amounts, confer a health benefit on the host^4^. This definition implies that probiotic status requires strain-specific characterisation and evidence of a clinically relevant health effect. Fermented foods, by contrast, are defined as ‘foods made through desired microbial growth and enzymatic conversions of food components^5^. Although many fermented foods such as yogurt and kefir may contain live microorganisms, not all fermented foods qualify as probiotics particularly those subjected to heat treatment (e.g., tarhana) or pasteurisation (e.g., commercially processed pickles), which may reduce or eliminate viable microbial populations.

In this context, microbiota awareness refers to an individual’s understanding of the role of the gut microbiota in health and recognition of behaviors that influence this balance. It also involves the ability to make informed dietary choices accordingly. Previous studies suggest that, particularly among young adults, knowledge about gut health does not always translate into healthy eating behaviors. Limited awareness may therefore be associated with lower consumption of probiotic and fermented foods^6,7^. University students are often considered a vulnerable group in this respect due to irregular meal patterns, time constraints, and increased reliance on convenience foods. Students enrolled in Faculties of Sport Sciences constitute a distinctive subgroup within the university population. Research has shown that higher levels of physical activity is associated with beneficial changes in gut microbiota composition. These effects include increased microbial diversity and enhanced production of short-chain fatty acids^8,9^. However, physical activity alone does not automatically lead to higher microbiota awareness or healthier dietary behaviors. Discrepancies between knowledge, attitudes, and actual practices may still exist.

Most existing research on probiotics and the gut microbiota has focused on clinical populations or the general public. In contrast, studies examining both microbiota awareness and probiotic or fermented food consumption among university students, particularly those studying sport sciences, remain limited. This gap is noteworthy, as these students are likely to become future coaches, physical education teachers, and sport scientists. Through these roles, they may indirectly influence public health. Therefore, assessing microbiota awareness levels among students of Faculties of Sport Sciences and exploring their relationship with probiotic food consumption may provide valuable insights. Based on existing literature suggesting that increased health-related awareness is generally associated with healthier behavioral choices, we hypothesized that higher levels of microbiota awareness would be positively associated with probiotic and fermented food consumption. The primary objective of this study was to examine the association between microbiota awareness and fermented food consumption among university students. As secondary objectives, the study also explored the relationships between microbiota awareness and selected sociodemographic characteristics, gastrointestinal symptoms, and dietary behaviors to provide a broader contextual understanding of factors associated with awareness and consumption patterns. Such findings could contribute to preventive health strategies and enrich the nutrition and performance literature in sport sciences. Ultimately, this study aims to establish a scientific basis for developing targeted educational and intervention programs for sport sciences students.

## Methods

### Study design and participants

This descriptive and analytical study was conducted with 435 university students (60.6% female, 39.4% male, aged 18–46, mean age 20.85 ± 3.51 years) between April and December 2025. Students from the Faculty of Sports Sciences at Aksaray University were included in the study. A priori power analysis was conducted using G*Power software (version 3.1.9.7) to estimate the required sample size. The analysis assumed a small effect size (f^2^ = 0.02), a significance level of α = 0.05, and a statistical power of 0.90. The minimum required sample size was calculated as 325 participants. To increase statistical robustness and account for potential missing or incomplete data, 435 participants were included in the final sample. Inclusion criteria were that university students should not have psychological illnesses that would prevent them from answering the survey questions, that they should be students studying in the Faculty of Sports Sciences, that they should not be pregnant or breastfeeding, and that they should be willing to participate in the study. Ethical approval was obtained from the Aksaray University Human Research Ethics Committee (Decision Number: 2025-129; Date: 18.03.2025). The study was conducted in accordance with the ethical principles stated in the Declaration of Helsinki. Written consent was obtained from the students who voluntarily participated in the study.

### Collection of data

University students who volunteered to participate in the study were given a questionnaire via Google Forms. The questionnaire consisted of 19 items covering sociodemographic characteristics, dietary habits, and health status.

*Microbiota Awareness Scale:* This scale was developed in 2020 by Özgür Önal and Aydan Külcü to assess microbiota awareness levels in individuals aged 18 and over who do not experience communication difficulties^10^. The scale, whose validity and reliability have been established, includes explanations of scientific terms used in the questions to help participants understand them more easily. The scale has sub-dimensions of general knowledge, probiotic and prebiotic nomenclature, chronic diseases, and product knowledge. High scores on the scale have been associated with a higher level of microbiota awareness.

*Food Consumption Frequency:* To assess habitual fermented food consumption, a Fermented Food Index (FFI) was created based on the frequency of consumption of commonly consumed conventional and commercially available fermented foods. The FFI was developed to capture overall dietary exposure to fermented foods commonly consumed in the Turkish population, rather than focusing on individual food items. The index was designed to reflect overall exposure to fermented foods rather than the consumption of individual products. Participants reported their usual frequency of consumption of the following fermented foods: homemade yogurt, probiotic-enriched yogurt, probiotic buttermilk, kefir, tarhana, cheese, table olives, boza, pickles, and turnip juice. These items were selected to represent a broad range of fermented foods commonly consumed in the Turkish population, including both foods likely to contain viable microorganisms at the point of consumption (e.g., homemade yogurt, probiotic-enriched yogurt, probiotic buttermilk, kefir) and traditionally fermented foods that may contain reduced or no viable microbial populations due to heat treatment or pasteurisation (e.g., tarhana, commercially processed pickles, shelf-stable olives). All items were included within a single composite index, and no distinction was made between these categories in the statistical analysis. The FFI was therefore treated as a measure of overall fermented food exposure rather than a direct measure of probiotic intake, and this should be considered when interpreting the findings.For each product, the frequency of consumption was assessed using a food frequency format with predefined response categories (never, once a month, once every 15 days, once or twice a week, three or four times a week, five or six times a week, once a day, twice a day, and three times a day)^11^. Each response category was converted into a numerical score representing the weekly frequency of consumption (scored from 0 = never to 8 = three times per day). This conversion allowed standardization of different consumption frequencies into a continuous metric suitable for statistical analysis. Next, scores for individual fermented food products were summed to create a total FFI score for each participant; higher scores indicated more frequent overall consumption of fermented foods. All items were equally weighted, as the aim of the index was to reflect overall fermented food consumption rather than the microbiological content or probiotic potency of individual foods. The Fermented Food Index demonstrated acceptable internal consistency (Cronbach’s α = 0.92), indicating coherence among items representing fermented food consumption frequency. However, it should be interpreted as a proxy measure of dietary exposure rather than a clinically validated measure of probiotic intake.

### Statistical analysis

Statistical analysis was performed using the Statistical Package for Social Sciences (version 23.0) (IBM Corp., Armonk, NY, USA). The normality of the data distribution was assessed using visual methods (histograms and probability plots) and analytical techniques (Shapiro–Wilk test). Descriptive statistics were presented as frequency and percentage for categorical variables, and as mean and standard deviation for numerical variables. Mann–Whitney U and Kruskal–Wallis tests were used for data that did not show a normal distribution. Relationships between numerical variables were presented using the Spearman correlation coefficient. Effect sizes were calculated and interpreted in accordance with established recommendations for non-parametric analyses. For Mann–Whitney U tests, the effect size was computed as r = Z/√N and interpreted using conventional thresholds of approximately 0.10 (small), 0.30 (medium), and 0.50 (large) effects^12,13^. For Kruskal–Wallis tests, epsilon squared (ε^2^) was calculated as ε^2^ = (H − k + 1)/(N − k) and interpreted as small (≈ 0.01), medium (≈ 0.06), and large (≈ 0.14) effects^14^.

To examine the independent association between microbiota awareness and fermented food consumption, a multivariate linear regression analysis was performed. Due to deviations from normality, the Fermented Food Index (FFI) was log-transformed prior to analysis. The model was adjusted for potential confounders, including age, BMI, sex, academic department, and parental education. Regression coefficients (B), 95% confidence intervals (CI), and *p*-values were reported. A *p*-value < 0.05 was considered statistically significant.

## Results

Table 1 shows the general characteristics of the sports science faculty students participating in the study, according to their department. 42.5% of the students are in coaching, 34.1% in physical education and sports teaching, and 23.4% in sports management.

**Table 1:** General characteristics of the participants (n = 435).

Variables	Coaching (n = 185)	Physical education and sports Teaching (n = 148)	Sports management (n = 102)
Age, years (M ± SD)	21.17 ± 3.10	20.62 ± 3.76	20.6 ± 3.8
Height, cm (M ± SD)	170.39 ± 10.05	168.39 ± 16.83	169.43 ± 9.72
BMI, kg/m^2^ (M ± SD)	23.17 ± 3.43	22.95 ± 3.88	22.71 ± 3.77
Gender, female, n (%)	106 (57.3)	91 (61.5)	67 (65.7)
Mother’s education n (%)			
No formal education	9 (4.9)	4 (2.7)	7 (6.9)
Primary school	68 (36.8)	62 (41.9)	43 (42.2)
Secondary school	37 (20.0)	34 (23.0)	14 (13.7)
High school	39 (21.1)	29 (19.6)	20 (19.6)
University	32 (17.3)	19 (12.8)	18 (17.6)
Father’s education n(%)			
No formal education	4 (2.2)	2 (1.4)	2 (2.0)
Primary school	54 (29.2)	34 (23.0)	35 (34.3)
Secondary school	37 (20.0)	33 (22.3)	25 (24.5)
High school	60 (32.4)	52 (35.1)	22 (21.6)
University	30 (16.2)	27 (18.2)	18 (17.6)
Chronic disease state, yes, n (%)	6 (3.2)	9 (6.2)	7 (6.8)
Allergy status, yes, n (%)	8 (4.3)	10 (6.9)	13 (12.8)
Drug use status, yes, n (%)	20 (10.8)	13 (8.8)	13 (12.7)
Experiencing constipation, yes, n (%)	22 (11.9)	11(7.4)	9 (8.8)
Diarrhea, yes, n (%)	47 (25.4)	43 (29.1)	26 (25.5)
Gastrointestinal gas complaints, n (%)			
Yes	23 (12.4)	14 (9.5)	10 (9.8)
Sometimes	52 (28.1)	47 (31.8)	29 (28.4)
Amount of water drunk per day			
2–4 glasses a day	42 (22.7)	31 (20.9)	38 (37.3)
5–7 glasses a day	61 (33.0)	59 (39.9)	25 (24.5)
8–10 glasses a day	51 (27.6)	37 (25.0)	23 (22.5)
11–12 glasses a day	31 (16.8)	21 (14.2)	16 (15.7)
Skipping main meals, yes, n (%)	155 (83.8)	121 (81.8)	84 (82.4)
The most frequently skipped meal n (%)			
Morning	76 (44.2)	63 (46.7)	41 (45.6)
Afternoon	64 (37.2)	44 (32.6)	38 (42.2)
Evening	13 (7.6)	10 (7.4)	4 (4.4)
Snack	19 (11.0)	18 (13.3)	7 (7.8)
Reason for skipping meals			
Lack of time	61 (35.3)	51 (38.1)	29 (31.2)
Not wanting to eat	57 (32.9)	44 (32.8)	39 (41.9)
Wanting to lose weight	15 (8.7)	2 (1.5)	9 (9.7)
Lack of habit	24 (13.9)	22 (16.4)	14 (15.1)
Because it is not prepared	14 (8.1)	12 (9.0)	2 (2.2)
Other	2 (1.2)	3 (2.2)	-
Frequency of vegetable and vegetable dish consumption n (%)			
Every day	18 (9.7)	11 (7.4)	5 (4.9)
4–5 days a week	61 (33.0)	54 (36.5)	50 (49.0)
Rarely	103 (55.7)	81 (54.7)	41 (40.2)
Never	3 (1.6)	2 (1.4)	6 (5.9)
Frequency of fruit consumption n (%)			
Every day	26 (14.1)	21 (14.2)	10 (9.8)
4–5 days a week	66 (35.7)	57 (38.5)	51 (50.0)
Rarely	85 (45.9)	68 (45.9)	40 (39.2)
Never	8 (4.3)	2 (1.4)	1 (1.0)
How to consume the fruit n (%)			
Fruit juice	26 (14.1)	13 (8.8)	13 (12.7)
Whole fruit (without peel)	90 (48.6)	62 (41.9)	48 (47.1)
Whole fruit (with peel)	69 (37.3)	73 (49.3)	41 (40.2)
Microbiota awareness scale (M ± SD)			
General information	19.42 ± 7.51	20.96 ± 6.77	18.98 ± 7.29
Probiotic and prebiotic	15.68 ± 5.94	16.49 ± 5.47	14.61 ± 5.45
Chronic disease	14.98 ± 5.64	15.83 ± 5.13	14.26 ± 5.20
Product information	9.36 ± 2.64	9.62 ± 2.34	8.84 ± 2.50
Total score	59.45 ± 19.53	62.91 ± 17.36	56.7 ± 17.7
Probiotic consumption status n (%)			
Yes	141 (76.2)	123 (83.1)	76 (74.5)
No	44 (23.8)	25 (16.9)	26 (25.5)
Reasons for not consuming probiotic products n (%)			
I find the use of probiotics unnecessary	15 (20.5)	2 (4.8)	6 (15.8)
I have no information about its importance	30 (41.1)	19 (45.2)	21 (55.3)
I don’t find it delicious	21 (28.8)	13 (31.0)	7 (18.4)
I can’t afford to buy probiotic products	7 (9.6)	8 (19.0)	4 (10.5)
Reasons for consuming probiotic products n (%)			
Due to health problems, upon expert advice (Dietitian/Doctor)	36 (22.6)	13 (10.8)	21 (25.0)
There is no health problem, and this is done on the advice of a specialist (Dietitian/Doctor)	38 (23.9)	28 (23.3)	24 (28.6)
Influenced by promotions related to probiotic foods (social media, television commercials, etc.)	42 (26.4)	43 (35.8)	21 (25.0)
Reading/following the literature related to the academic studies conducted	32 (20.1)	25 (20.8)	16 (19.0)
Other	11 (6.9)	11 (9.2)	2 (2.4)
Believing that the disease and/or its symptoms have decreased or completely disappeared due to the consumption of probiotic products n (%)			
Yes, I think so myself	108 (58.4)	83 (56.1)	7 (46.1)
Yes, expert opinion (Dietitian/Doctor)	25 (13.5)	15 (10.1)	20 (19.6)
I didn’t see any benefit	52 (28.1)	50 (33.8)	35 (34.3)

Table 2 presents the sub-dimensions of the microbiota awareness scale and various variables among students studying in the Faculty of Sports Sciences. Female students showed significantly higher scores than male students in product knowledge, probiotic-prebiotic knowledge, and total microbiota awareness (*p* < 0.05; r = 0.11–0.20), whereas no significant differences were observed in general knowledge or chronic disease subscales. Regarding departmental differences, students in the Physical Education and Sports Teaching program scored higher in product knowledge, probiotic-prebiotic awareness, and total scores compared to Sports Management students (*p* < 0.05; ε^2^ = 0.009–0.015). Similarly, microbiota awareness differed across BMI categories; underweight, overweight, and obese students demonstrated higher total scores than those with normal BMI (*p* = 0.029; ε^2^ = 0.014).

**Table 2:** Evaluation of microbiota awareness among students studying at the Faculty of Sports Sciences according to certain variables.

	General information M ± SD	Product information M ± SD	Chronic disease M ± SD	Probiotic and prebiotic M ± SD	Total score M ± SD
Gender					
Female	20.46 ± 6.78	9.64 ± 2.46	15.34 ± 5.01	16.27 ± 5.37	61.72 ± 17.42
Male	18.87 ± 7.83	8.84 ± 2.53	14.74 ± 5.93	14.84 ± 6.09	57.30 ± 19.81
*p*	0.074^a^	** < 0.001**^**a**^	0.297^a^	**0.018**^**a**^	**0.026**^**a**^
Effect size	r = -0.086	r = -0.16	r = -0.05	r = -0.11	r = -0.11
Department					
Coaching	19.42 ± 7.51	9.36 ± 2.64	14.98 ± 5.64	15.68 ± 5.94	59.45 ± 19.53
Physical education and sports teaching	20.96 ± 6.77	9.62 ± 2.34	15.83 ± 5.13	16.49 ± 5.47	62.91 ± 17.36
Sports management	18.98 ± 7.29	8.84 ± 2.50	14.26 ± 5.20	14.61 ± 5.45	56.70 ± 17.70
*p*	0.067^b^	**0.049**^**b**^	**0.046**^**b**^	**0.026**^**b**^	**0.019**^**b**^
Effect size	ε^2^ = 0.008	ε^2^ = 0.009	ε^2^ = 0.010	ε^2^ = 0.012	ε^2^ = 0.014
BMI, kg/m^2^					
Underweight	20.59 ± 6.51	9.33 ± 2.55	15.35 ± 5.20	16.09 ± 5.48	61.38 ± 17.55
Normal	19.15 ± 7.30	9.31 ± 2.51	14.57 ± 5.30	15.12 ± 5.63	58.16 ± 18.34
Overweight	21.28 ± 7.21	9.29 ± 2.53	16.20 ± 5.64	16.80 ± 5.83	63.58 ± 18.94
Obese	20.47 ± 7.34	9.76 ± 2.70	16.52 ± 4.83	17.58 ± 5.63	64.35 ± 18.13
*p*	**0.025**^**b**^	**0.026**^**b**^	**0.025**^**b**^	0.735^b^	**0.029**^**b**^
Effect size	ε^2^ = 0.015	ε^2^ = 0.015	ε^2^ = 0.015	ε2 = − 0.004	ε^2^ = 0.014
Experiencing constipation					
Yes	21.59 ± 6.72	10.02 ± 2.72	16.38 ± 4.96	17.33 ± 5.74	65.33 ± 17.99
No	19.65 ± 7.28	9.25 ± 2.49	14.97 ± 5.42	15.71 ± 5.70	59.41 ± 18.49
*p*	0.142^a^	0.113^a^	0.080^a^	**0.033**^**a**^	**0.034**^**a**^
Effect size	r = 0.07	r = 0.08	r = 0.08	r = 0.10	r = 0.10
Diarrhea					
Yes	21.20 ± 6.70	9.56 ± 2.45	15.75 ± 5.19	16.34 ± 5.32	62.86 ± 17.23
No	19.34 ± 7.38	9.24 ± 2.54	14.87 ± 5.45	15.47 ± 5.82	58.94 ± 18.86
*p*	**0.017**^**a**^	0.273^a^	0.228^a^	0.162^a^	0.060^a^
Effect size	r = 0.12	r = 0.05	r = 0.06	r = 0.07	r = 0.09
Gastrointestinal gas complaints					
Yes	19.25 ± 7.64	9.70 ± 2.47	14.91 ± 5.15	15.40 ± 5.70	59.27 ± 18.67
No	19.10 ± 7.43	9.06 ± 2.54	14.76 ± 5.66	15.25 ± 5.88	58.18 ± 19.22
Sometimes	21.57 ± 6.45	9.71 ± 2.45	15.88 ± 4.84	16.75 ± 5.22	63.92 ± 16.37
*p*	**0.005**^**b**^	**0.029**^**b**^	0.295^b^	0.091^b^	**0.020**^**b**^
Effect size	ε^2^ = 0.020	ε^2^ = 0.012	ε^2^ = 0.001	ε^2^ = 0.007	ε^2^ = 0.013
Skipping main meals					
Yes	19.92 ± 7.14	9.45 ± 2.46	15.16 ± 5.23	15.83 ± 5.63	60.37 ± 18.19
No	19.45 ± 7.77	8.73 ± 2.74	14.82 ± 6.13	15.13 ± 6.05	58.14 ± 19.96
*p*	0.678^a^	0.071^a^	0.691^a^	0.430^a^	0.372^a^
Effect size	r = 0.02	r = 0.09	r = 0.02	r = 0.04	r = 0.04
The most frequently skipped meal					
Morning	19.61 ± 7.46	9.27 ± 2.61	15.18 ± 5.45	15.53 ± 5.84	59.6 ± 19.07
Afternoon	20.25 ± 6.57	9.59 ± 2.37	15.23 ± 4.88	16.23 ± 5.07	61.32 ± 16.57
Evening	20.11 ± 7.59	8.62 ± 2.77	15.74 ± 6.09	15.92 ± 6.38	60.40 ± 20.43
Snack	20.45 ± 7.75	9.18 ± 2.64	15.34 ± 5.98	16.36 ± 6.23	61.34 ± 20.43
*p*	0.913^b^	0.451^b^	0.907^b^	0.637^b^	0.856^b^
Effect size	ε^2^ = 0.00	ε^2^ = 0.00	ε^2^ = 0.00	ε^2^ = 0.00	ε^2^ = 0.00
Reason for skipping meals					
Lack of time	19.01 ± 7.63	9.17 ± 2.69	14.63 ± 5.56	15.40 ± 5.83	58.21 ± 19.25
Not wanting to eat	20.67 ± 7.22	8.99 ± 2.47	15.73 ± 5.34	16.11 ± 5.64	61.52 ± 18.45
Wanting to lose weight	19.23 ± 6.63	9.38 ± 1.98	14.88 ± 4.39	16.15 ± 5.40	59.65 ± 16.79
Lack of habit	20.13 ± 6.55	9.95 ± 2.23	15.05 ± 5.46	15.81 ± 5.70	60.95 ± 17.75
Because it is not prepared	20.21 ± 7.17	10.25 ± 2.97	15.64 ± 5.45	16.21 ± 5.64	62.32 ± 18.88
Other	22.00 ± 5.70	10.00 ± 1.41	16.00 ± 3.93	17.6 ± 4.77	65.60 ± 14.43
*p*	0.390^b^	0.111^b^	0.857^b^	0.857^b^	0.601^b^
Effect size	ε^2^ = 0.001	ε^2^ = 0.009	ε^2^ = 0.00	ε^2^ = 0.00	ε^2^ = 0.00
Frequency of vegetable and vegetable dish consumption					
Every day	17.47 ± 7.19	8.29 ± 2.55	14.61 ± 5.59	14.79 ± 5.62	55.17 ± 19.12
4–5 days a week	20.21 ± 7.33	9.72 ± 2.57	15.28 ± 5.51	15.77 ± 5.81	61.00 ± 18.77
Rarely	20.21 ± 6.99	9.22 ± 2.46	15.26 ± 5.12	16.04 ± 5.48	60.75 ± 17.66
Never	14.09 ± 8.46	8.72 ± 1.84	10.72 ± 7.00	10.63 ± 6.74	44.18 ± 22.42
*p*	**0.010**^**b**^	**0.027**^**b**^	0.093^b^	**0.040**^**b**^	**0.027**^**b**^
Effect size	ε^2^ = 0.019	ε^2^ = 0.014	ε^2^ = 0.008	ε^2^ = 0.012	ε^2^ = 0.014
Frequency of fruit consumption					
Every day	17.57 ± 8.24	8.96 ± 2.69	14.22 ± 6.37	14.15 ± 6.47	54.92 ± 21.31
4–5 days a week	20.25 ± 7.13	9.52 ± 2.58	15.41 ± 5.26	16.22 ± 5.55	61.43 ± 18.21
Rarely	20.29 ± 6.93	9.29 ± 2.42	15.18 ± 5.17	15.8 ± 5.54	60.57 ± 17.64
Never	17.27 ± 7.33	8.63 ± 2.41	13.36 ± 5.69	13.90 ± 5.55	53.18 ± 19.08
*p*	0.117^b^	0.404^b^	0.498^b^	0.153^b^	0.161^b^
Effect size	ε^2^ = 0.007	ε^2^ = 0.00	ε^2^ = 0.00	ε^2^ = 0.005	ε^2^ = 0.005
How to consume the fruit					
Fruit juice	17.61 ± 7.65	8.44 ± 2.63	13.25 ± 5.68	14.32 ± 6.09	53.63 ± 20.31
Whole fruit (without peel)	20.19 ± 6.89	9.45 ± 2.47	15.24 ± 5.05	15.90 ± 5.42	60.8 ± 17.26
Whole fruit (with peel)	20.09 ± 7.43	9.44 ± 2.50	15.48 ± 5.59	15.89 ± 5.86	60.91 ± 19.03
*p*	0.072^b^	**0.028**^**b**^	**0.045**^**b**^	0.196^b^	**0.044**^**b**^
Effect size	ε^2^ = 0.007	ε^2^ = 0.012	ε^2^ = 0.009	ε^2^ = 0.002	ε^2^ = 0.009
Probiotic consumption status					
Yes	20.2 ± 7.16	9.58 ± 2.49	15.37 ± 5.25	16.1 ± 5.67	61.27 ± 18.08
No	18.56 ± 7.46	8.40 ± 2.42	14.13 ± 5.80	14.28 ± 5.61	55.38 ± 19.35
*p*	**0.024**^**a**^	** < 0.001**^**a**^	**0.044**^**a**^	**0.003**^**a**^	**0.002**^**a**^
Effect size	r = 0.11	r = 0.20	r = 0.10	r = 0.15	r = 0.15
Reasons for not consuming probiotic products					
I find the use of probiotics unnecessary	13.65 ± 6.78	7.21 ± 2.64	11.04 ± 5.47	11.30 ± 5.32	43.21 ± 18.47
I have no information about its importance	19.55 ± 6.90	8.02 ± 2.62	15.11 ± 5.46	14.91 ± 5.30	57.61 ± 18.08
I don’t find it delicious	17.12 ± 7.99	8.75 ± 2.14	13.21 ± 5.88	13.60 ± 6.15	52.70 ± 20.26
I can’t afford to buy probiotic products	20.10 ± 9.07	8.15 ± 2.14	15.89 ± 7.31	16.21 ± 6.45	60.36 ± 22.58
*p*	**0.007**^**b**^	0.112^b^	**0.019**^**b**^	**0.044**^**b**^	**0.019**^**b**^
Effect size	ε^2^ = 0.021	ε^2^ = 0.007	ε^2^ = 0.016	ε^2^ = 0.012	r = 0.15
Reasons for consuming probiotic products					
Due to health problems, upon expert advice (Dietitian/Doctor)	17.77 ± 7.94	8.52 ± 2.75	14.05 ± 5.73	14.41 ± 6.18	54.77 ± 20.71
There is no health problem, and this is done on the advice of a specialist (Dietitian/Doctor)	19.22 ± 7.17	9.33 ± 2.70	14.71 ± 5.38	15.41 ± 5.82	58.67 ± 18.61
Influenced by promotions related to probiotic foods (social media, television commercials, etc.)	20.37 ± 7.32	9.71 ± 2.21	15.20 ± 5.39	16.16 ± 5.50	61.47 ± 18.25
Reading/following the literature related to the academic studies conducted	20.41 ± 7.32	9.82 ± 2.57	15.84 ± 5.45	16.72 ± 5.92	62.80 ± 18.49
Other	21.54 ± 6.18	9.45 ± 2.85	15.00 ± 4.55	15.41 ± 5.77	61.41 ± 15.90
*p*	0.152^b^	**0.037**^**b**^	0.606^b^	0.244^b^	0.253^b^
Effect size	ε^2^ = 0.006	ε^2^ = 0.014	ε^2^ = 0.00	ε^2^ = 0.003	ε^2^ = 0.003
Believing that the disease and/or its symptoms have decreased or completely disappeared due to the consumption of probiotic products					
Yes, I think so myself	20.21 ± 7.39	9.38 ± 2.53	15.61 ± 5.51	16.34 ± 5.91	61.55 ± 18.90
Yes, expert opinion (Dietitian/Doctor)	18.01 ± 7.54	8.33 ± 3.13	13.98 ± 5.48	14.68 ± 5.63	55.01 ± 19.36
I didn’t see any benefit	20.00 ± 6.78	9.66 ± 2.06	14.72 ± 5.06	15.05 ± 5.23	59.45 ± 17.09
*p*	0.062^b^	**0.034**^**b**^	**0.024**^**b**^	**0.006**^**b**^	**0.013**^**b**^
Effect size	ε^2^ = 0.008	ε^2^ = 0.011	ε^2^ = 0.013	ε^2^ = 0.019	ε^2^ = 0.016

Values are presented as mean ± standard deviation. *p* < 0.05 was considered statistically significant. ^a^ Mann Whitney U test, ^b^Kruskal-Wallis test, Effect sizes were calculated as r = Z/√N for Mann–Whitney U tests and ε^2^ = (H − k + 1)/(N − k) for Kruskal–Wallis tests.

Students reporting constipation showed higher probiotic-prebiotic and total awareness scores compared to those without constipation (*p* < 0.05; r = 0.10–0.15). Likewise, participants experiencing diarrhea had higher general knowledge scores (*p* = 0.017; r = 0.12). Gastrointestinal gas complaints were associated with differences in general knowledge, product knowledge, and total scores (ε^2^ = 0.010–0.020; *p* < 0.05).

Dietary habits were associated with microbiota awareness. Daily vegetable consumption was associated with total awareness scores compared with consumption 4–5 days per week (*p* = 0.027; ε^2^ = 0.014), while no significant associations were observed for students who did not consume vegetables across subscales. Fruit consumption frequency was not significantly associated with awareness scores (*p* > 0.05; ε^2^ = 0.005–0.008); however, fruit juice consumption was associated with lower product knowledge, probiotic-prebiotic, and total scores compared with whole fruit consumption (*p* < 0.05; r = 0.05–0.15).

Students consuming probiotic products had significantly higher scores across all subscales and total microbiota awareness compared with non-consumers (*p* ≤ 0.044; r = 0.10–0.20). Among non-consumers, those who considered probiotics unnecessary showed significantly lower awareness scores (*p* = 0.019; ε^2^ = 0.014). Additionally, students who reported that probiotics alleviated symptoms based on personal experience or expert opinion demonstrated higher probiotic-prebiotic and total awareness scores (*p* < 0.05; ε^2^ = 0.013–0.019).

Correlation analysis revealed strong and significant positive relationships among the sub-dimensions of the Microbiota Awareness Scale (MAS) (Table 3). General knowledge showed a strong correlation with probiotic-prebiotic knowledge (r = 0.845, *p* < 0.01) and chronic disease awareness (r = 0.822, *p* < 0.01). Similarly, the probiotic-prebiotic and chronic disease subscales also showed a high correlation (r = 0.871, *p* < 0.01).

**Table 3:** Spearman Correlation Analysis Between MAS Subdimensions and Fermented Food Intake Index (FFI).

Variables	1	2	3	4	5	6
1. General information	1.000	0.845**	0.822**	0.252**	0.929**	− 0.278**
2. Probiotic-prebiotic	0.845**	1.000	0.871**	0.244**	0.942**	− 0.195**
3. Chronic disease	0.822**	0.871**	1.000	0.173**	0.915**	− 0.164**
4. Product information	0.252**	0.244**	0.173**	1.000	0.388**	− 0.207**
5. Total MAS score	0.929**	0.942**	0.915**	0.388**	1.000	− 0.234**
6. Total FFI index	− 0.278**	− 0.195**	− 0.164**	− 0.207**	− 0.234**	1.000

**Correlation is significant at the 0.01 level (2-tailed).

The total MAS score was strongly associated with all sub-dimensions, especially probiotic-prebiotic (r = 0.942, *p* < 0.01), general knowledge (r = 0.929, *p* < 0.01), and chronic disease awareness (r = 0.915, *p* < 0.01). Product knowledge showed weaker but still significant correlations with other MAS components (r = 0.173–0.388, *p* < 0.01). In contrast, the total Fermented Food Index (FFI) score showed a negative correlation with all MAS sub-dimensions and the total MAS score. The strongest inverse relationship was observed between FFI and general knowledge (r =  − 0.278, *p* < 0.01), followed by the total MAS score (r =  − 0.234, *p* < 0.01).

A multivariate linear regression analysis was conducted using log-transformed FFI scores to account for non-normal distribution. After adjusting for age, BMI, sex, academic department, and parental education, microbiota awareness remained significantly and inversely associated with fermented food consumption (B =  − 0.008, 95% CI − 0.010 to − 0.005, *p* < 0.001). None of the covariates showed a statistically significant association with the outcome.

**Fig. 1:**
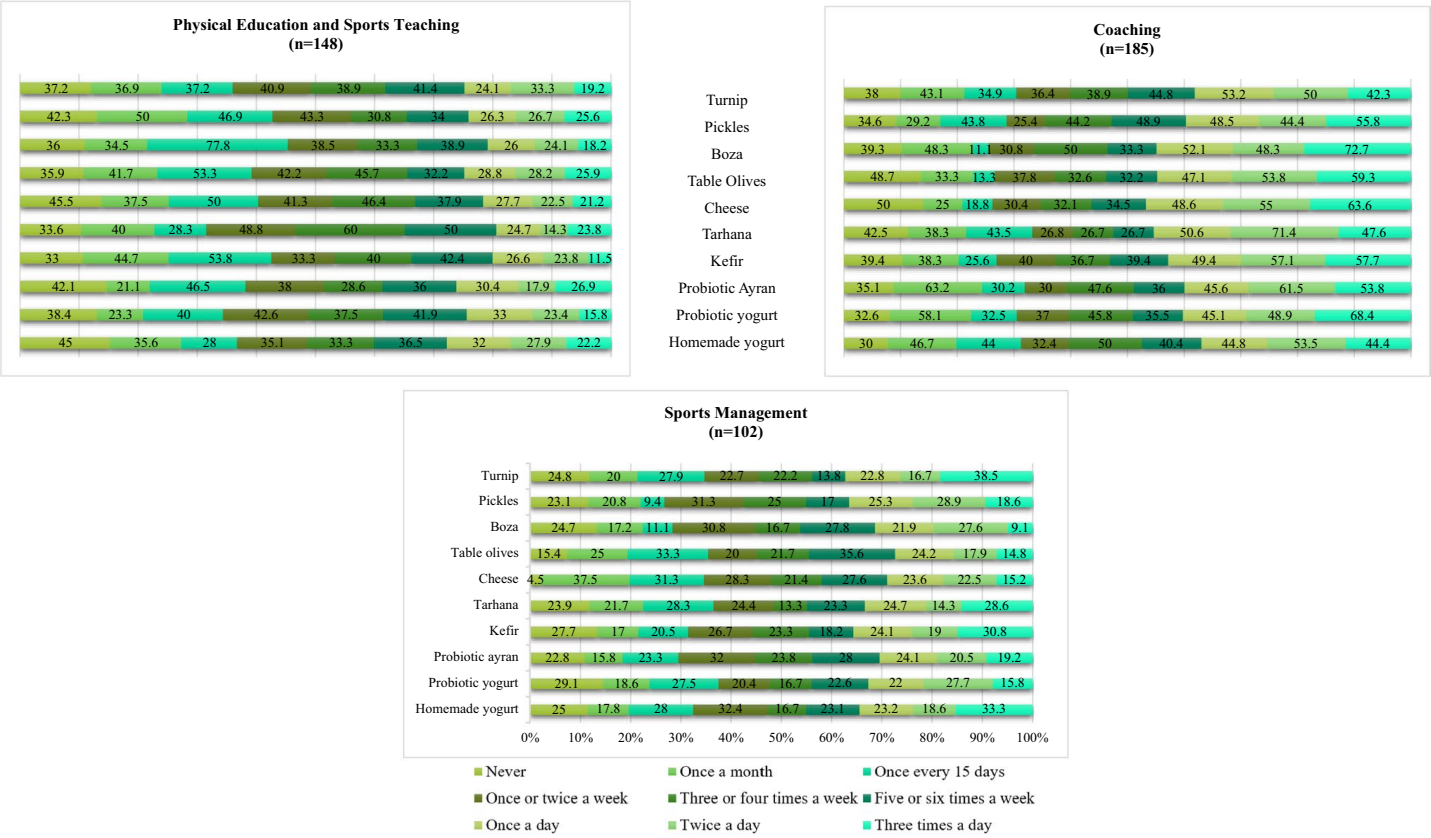
Fermented food consumption frequencies according to department.

Figure 1 presents the distribution of fermented food consumption frequencies according to academic department. Students in the Physical Education and Sports Teaching and Coaching departments reported higher consumption frequencies for most fermented foods, particularly probiotic yogurt, probiotic ayran, kefir, and homemade yogurt, compared with students in Sports Management. In contrast, Sports Management students more frequently reported low consumption levels (never or once a month) across several fermented food categories. Traditional fermented foods such as tarhana, table olives, and cheese were consumed more regularly in the Coaching department, whereas the highest proportions of frequent consumption (five or more times per week) for probiotic products were observed among Physical Education and Sports Teaching students.

## Discussion

Investigating microbiota awareness among students in sports science faculties is important because this group represents a population with high levels of physical activity, increased nutritional needs, and a growing interest in performance and health-oriented nutrition practices. Understanding microbiota awareness in this context can provide valuable insights into how future coaches, teachers, and sports professionals perceive gut health and how they translate scientific knowledge into nutritional behaviors that can impact both personal health and professional practice. Awareness of the impact of microorganisms, particularly the microbiota, on human health is extremely important for individuals, and especially athletes. This awareness may be associated with personal lifestyle decisions and may inform policymakers in developing strategies^15^.

The general characteristics of students across departments indicate a largely homogeneous sample in terms of age, BMI, and anthropometric measures, reflecting the expected profile of physically active university students in sports science programs (Table 1). Mean BMI values remained within the normal range across all groups, which is consistent with evidence linking regular physical activity to more favorable body composition in young adults^16^. Despite a generally healthy profile, dietary habits such as skipping meals and low consumption of fruits and vegetables were observed, particularly among Sports Management students. Such patterns are commonly reported among university students and persist even in physically active populations, potentially negatively impacting long-term metabolic and gut health^17^. Furthermore, the relatively high frequency of gastrointestinal complaints despite young age and good health status is noteworthy and may be associated with irregular eating habits, fluid intake practices, and exercise-related stress^18^. While probiotic consumption, as shown in Table 1, is prevalent, particularly in education-focused departments, decisions to consume probiotics are often influenced by social media or non-academic sources, with a lack of information remaining the primary reason for non-consumption. This finding underscores a critical gap between health-focused education and evidence-based nutritional behavior, even among future sports professionals, and provides important context for interpreting microbiota awareness outcomes in this population (Table 4).

**Table 4:** Multivariate linear regression analysis of factors associated with fermented food consumption (log-transformed).

Variable	B	β	95% CI	p-value
Microbiota awareness (MAS)	− 0.008	− 0.318	(− 0.010, − 0.005)	< 0.001
Age	0.010	0.083	(− 0.001, 0.022)	0.082
BMI	0.006	0.049	(− 0.006, 0.017)	0.310
Sex	− 0.068	− 0.075	(− 0.154, 0.018)	0.123
Department	− 0.043	− 0.077	(− 0.094, 0.007)	0.090
Mother’s education	0.008	0.021	(− 0.030, 0.045)	0.681
Father’s education	0.000	− 0.001	(− 0.040, 0.040)	0.982

Adjusted for age, BMI, sex, academic department, and parental education.

Sex, academic background, gastrointestinal experience, and probiotic-related beliefs emerged as the primary correlates of microbiota awareness in this sample, whereas general dietary habits showed limited and inconsistent associations. Female students demonstrated higher scores in product knowledge, probiotic-prebiotic knowledge, and total microbiota awareness (*p* < 0.05); however, effect sizes were small, indicating limited practical relevance (Table 2).This pattern aligns with previous evidence indicating higher nutrition literacy and health-related information seeking behaviors among women^19,20^. The differences observed among academic departments suggest that curriculum-related experience may be associated with differences in microbiota awareness. The higher awareness scores of students in Physical Education and Sports Teaching departments compared to those in Sports Management departments may reflect a greater emphasis on health, physiology, and nutrition content in teacher and coach-focused programs. Similar associations between academic exposure and nutrition knowledge have been documented in university populations^21^. Although differences across academic departments and BMI categories were statistically significant, effect sizes were consistently small (ε^2^ = 0.01–0.02), indicating limited practical relevance of these associations in explaining variability in microbiota awareness. A particularly noteworthy finding is that students reporting gastrointestinal symptoms such as constipation, diarrhea, or occasional gas have higher awareness of the microbiota (Table 2). This relationship may be explained by a symptom-focused learning pattern rather than a protective effect of awareness; in this process, individuals experiencing gastrointestinal discomfort may be more likely to seek information about concepts related to gut health, probiotics, and the microbiota. This interpretation is supported by recent studies highlighting the bidirectional relationship between gastrointestinal symptoms and the acquisition of health knowledge^22,23^. An alternative explanation for the observed relationships is the possibility of reverse causality. For example, individuals experiencing gastrointestinal symptoms or having lower consumption of fermented foods may be more likely to seek information about gut health, which could increase their microbiota awareness. This interpretation is supported by recent evidence showing that health information-seeking behavior is triggered by perceived health needs, symptoms, and contextual factors rather than solely by previously acquired information^24,25^. Specifically, a meta-analytic review has shown that health status and perceived needs are key drivers of information-seeking behavior, suggesting that individuals may actively seek information in response to current conditions, rather than as a precursor to behavioral change. Given the cross-sectional design in the present study, it is not possible to determine the direction of these relationships.

Although statistically significant relationships were observed, the relatively small differences in awareness scores among nutritional behavior groups suggest that microbiota awareness may not directly translate to adherence to specific dietary recommendations. Instead, these patterns point to a closer link between microbiota awareness and overall health consciousness than to specific eating behaviors. Similar knowledge-behavior gaps have been noted in studies examining nutritional knowledge and diet quality among young adults^26,27^. Probiotic consumption status emerged as one of the strongest correlates of microbiota awareness. Students who consumed probiotics or believed in their symptom-relieving effects demonstrated higher awareness across multiple sub-dimensions, whereas those who perceived probiotics as unnecessary had the lowest scores. This finding underscores persistent misconceptions and information gaps regarding probiotics, even among physically active and health-oriented student groups, and highlights the need for evidence-based nutrition education focusing on gut health.

Table 3 evaluates the relationships between MAS sub-dimensions and FFI. The MAS sub-dimension, general knowledge, showed a high correlation with both probiotic-prebiotic knowledge and chronic disease awareness. The strong relationship between probiotic-prebiotic knowledge and chronic disease awareness suggests that students relate microbiota concepts not only to nutrition but also to broader health-related outcomes. This is consistent with existing evidence highlighting the role of gut microbiota in immune regulation, metabolic processes, and chronic disease mechanisms^2,7^. As expected, the total MAS score was strongly correlated with all sub-dimensions, with the highest correlations observed for probiotic-prebiotic knowledge, general knowledge, and chronic disease awareness. In this sample, these findings point to these three areas as the main components associated with microbiota awareness. The very high intercorrelations among MAS sub-dimensions (r > 0.80) suggest substantial conceptual overlap and raise concerns regarding discriminant validity. This pattern may indicate that the sub-dimensions reflect closely related facets of a broader latent construct rather than fully distinct dimensions^28,29^.

This interpretation is supported by previous research showing that probiotics and fermented foods have been reported to be associated with gut health and immune and metabolic regulation in previous studies. This may also be associated with increased health-related awareness as scientific knowledge in these areas expands^3^. In contrast, product knowledge showed weaker correlations with other MAS components. This is an important observation because it may indicate that while students appear to demonstrate a conceptual understanding of microbiota and health, they may encounter difficulties in applying this knowledge to daily consumer decisions, such as choosing probiotic-labeled products. Similar application gaps have been documented in young adult populations, where food-related choices are shaped by marketing exposure, peer influence, and non-scientific information sources^6^. One of the notable findings of this study is the weak negative correlation between FFI and MAS scores. Although this direction was not strongly specified a priori, it aligns with previous literature suggesting that increased health-related awareness does not always translate into corresponding dietary behaviors. Instead of showing that fermented food consumption increases with increased awareness, the results indicate a gap between knowledge and actual behavioral application. This discrepancy can be more comprehensively understood through a behavioural science perspective, particularly within the framework of the intention–action gap. While higher awareness may reflect knowledge and favourable attitudes towards gut health, it does not necessarily lead to consistent dietary behaviour. According to established models such as the Health Belief Model^30^ and the Theory of Planned Behavior^31^, behaviour is shaped not only by knowledge but also by perceived barriers, subjective norms, and perceived behavioural control. In a university setting, practical constraints such as limited time, financial considerations, food availability, and established eating habits may hinder the translation of awareness into action. To support this statement, previous studies have reported that nutritional knowledge does not always translate into healthier eating behaviors among young adult groups^26,27^. To further interpret this counterintuitive finding, several contextual and behavioural factors should be considered. First, economic and accessibility barriers may limit students’ ability to regularly consume fermented or probiotic-rich foods, particularly commercially available products such as kefir or probiotic yogurt. Previous research has shown that structural and environmental factors, including cost and availability, play an important role in shaping dietary choices among young adults^26,27^. Sensory and taste-related factors may further compound this: fermented products with characteristically sour, sharp, or unfamiliar flavour profiles such as kefir, boza, or turnip juice may be avoided by younger consumers regardless of their health-related beliefs, as food acceptance is driven not only by nutritional knowledge but also by hedonic and sensory preferences^32^. Second, cultural dietary habits may influence consumption patterns, as traditional fermented foods are not always perceived as functional or health-promoting despite their potential microbiota-related benefits^3^. In the Turkish dietary context, foods such as homemade yogurt, tarhana, and table olives are consumed as everyday staples rather than as deliberate health interventions, which may reduce their perceived relevance to microbiota health even among individuals with high awareness. This cultural normalisation of fermented food consumption may paradoxically weaken the link between gut health knowledge and conscious dietary behaviour^33^. Third, exposure to non-scientific information sources and inconsistent health messages may lead individuals to associate gut health primarily with commercially labeled probiotic products rather than everyday dietary practices. This distinction is rarely communicated clearly in lay health media, and its absence may give rise to cognitive dissonance: students who hold high awareness of microbiota health may simultaneously perceive their diet as adequate by virtue of occasional commercial probiotic use, while undervaluing or overlooking the contribution of traditional fermented foods to microbial diversity^34^. Finally, this finding highlights a discrepancy between theoretical awareness and its practical application, suggesting that knowledge alone may not be sufficient to drive consistent dietary behavior, as also emphasized in behavioral nutrition literature^26,27^. Furthermore, it should be acknowledged that the FFI captures fermented food exposure broadly and does not differentiate between products containing viable microorganisms and those that do not, due to heat treatment or pasteurisation. This methodological limitation may have attenuated or obscured any potential association between microbiota awareness and true probiotic intake. Therefore, the observed inverse association should be interpreted with caution, and future studies employing more precise measures of probiotic exposure are warranted.

Figure 1 complements these findings by illustrating students’ consumption patterns across a range of fermented and probiotic foods, including traditional items such as pickles, turnip juice, boza, tarhana, table olives, cheese, and homemade yogurt, alongside products that are more commonly marketed as probiotics (e.g., probiotic yogurt, probiotic ayran) and kefir. This distribution suggests that fermented food intake may be influenced both by cultural dietary habits and by contemporary health trends. However, it also highlights the importance of improving nutrition literacy in a way that clearly distinguishes between traditional fermented foods and probiotic products with scientifically supported effects^3^. It is also worth noting that some frequently consumed items, such as tarhana and commercially processed pickles, may not retain viable microbial populations due to heat treatment or pasteurisation. Therefore, high consumption frequencies for these products may not reflect meaningful probiotic exposure, and overall FFI scores should be interpreted accordingly. Overall, these results reinforce the need for practice-oriented educational strategies that extend beyond information provision and incorporate behaviour change techniques such as goal setting, self-monitoring, and guided food-choice training to help students translate microbiota awareness into sustainable dietary habits^6^. Improving accessibility to diverse fermented food options within university food environments and supporting students in critically interpreting nutritional labelling may also help bridge the observed gap between awareness and behaviour. As sports science graduates move into professional roles where they will advise and influence physically active populations, the quality of their gut health literacy and its practical application carries implications beyond personal health.

This study demonstrates that microbiota awareness among sports science students is shaped primarily by academic background, sex, gastrointestinal experiences, and probiotic-related beliefs rather than by general dietary habits alone. While overall awareness was relatively strong especially in key theoretical domains such as general knowledge, probiotic–prebiotic knowledge, and chronic disease awareness these gains were not reflected in higher fermented food consumption, pointing to a persistent dissociation between theoretical knowledge and dietary practice. As sports science graduates move into professional roles advising physically active populations, the quality of their gut health literacy carries implications beyond personal health.Therefore, incorporating evidence-based and practice-oriented gut health education into sports science curricula may help improve consumer decision-making, reduce misconceptions about probiotics, and support the translation of microbiota awareness into sustainable dietary behaviors.

## Conclusion

The findings of this study suggest that increased awareness of the gut microbiota does not necessarily translate into healthier dietary practices, even among individuals who are physically active and expected to possess relatively high health literacy. The dissociation between awareness and fermented food intake observed in the present sample reflects a broader pattern documented in behavioural nutrition literature, in which knowledge serves as a necessary but insufficient condition for dietary change. The higher awareness levels reported by students experiencing gastrointestinal symptoms further suggest that microbiota awareness may be higher among individuals reporting gastrointestinal symptoms, rather than as part of a preventive dietary mindset. From a practical perspective, these results emphasise the need for nutrition education strategies that extend beyond information provision and incorporate behavior change techniques such as goal setting, self-monitoring, and guided food-choice training. Improving accessibility to fermented food options within university food environments and supporting students in interpreting nutritional information may help bridge the gap between awareness and dietary behavior. Given the exploratory nature of the study and multiple subgroup comparisons, no formal adjustment for multiple testing was applied. However, effect sizes were reported alongside p-values to mitigate the risk of Type I error and to provide a more robust interpretation of the findings. Overall, effect size estimates indicate that although several associations reach statistical significance, their magnitude is generally small. Given the cross-sectional design of this study, all observed relationships should be interpreted as associations rather than causal effects.

## Strengths and limitations

A key strength of this study is its focus on the gap between microbiota awareness and actual dietary behaviour, an area that remains underexplored despite increasing public interest in gut health. The inclusion of fermented food consumption alongside awareness and knowledge measures provides a more comprehensive understanding of how microbiota-related concepts are reflected in everyday eating practices. In addition, the study sample comprised sports science students, a group expected to demonstrate relatively high health literacy, allowing for the examination of awareness–behaviour discrepancies even within a theoretically advantaged population.

Several limitations should also be acknowledged. The cross-sectional design precludes any causal interpretation of the observed associations. All data were self-reported, which may introduce recall or social desirability bias. In particular, the retrospective nature of food frequency reporting may lead participants to over- or underestimate their habitual fermented food consumption, which could attenuate or distort the observed associations between dietary behaviour and microbiota awareness. Furthermore, the study was conducted at a single institution, limiting the generalisability of the findings to other student populations or age groups. Another important limitation concerns the structure of the Fermented Food Index (FFI). The index is based primarily on consumption frequency and does not account for portion sizes, which may lead to imprecision in estimating actual intake levels. In addition, fermented food items were treated with equal weighting in the FFI, although these foods may differ in terms of microbial content, viability, and potential health effects. The FFI also does not capture the viability or quantity of probiotic microorganisms, nor does it differentiate between fermented foods that are likely to contain viable microorganisms at the point of consumption (e.g., kefir, homemade yogurt) and those that may not, due to heat treatment or pasteurisation during processing (e.g., tarhana, commercially processed pickles). Therefore, the FFI may not fully reflect the true physiological impact of fermented food consumption, and the findings should be interpreted with caution. Although the FFI demonstrated high internal consistency, this does not establish construct validity, and the index should be interpreted as a proxy of dietary patterns rather than a direct measure of probiotic intake. Additionally, the high correlations observed between MAS sub-dimensions suggest potential construct overlap, which may limit the distinct interpretation of individual subscale scores. As probiotic consumption status was assessed as part of a broader dietary habits questionnaire, individual differences in the interpretation of the term ‘probiotic’ may have introduced some variability in self-reported data. This is acknowledged as a limitation, though it does not affect the FFI, which was designed to capture overall fermented food exposure rather than clinically verified probiotic intake. The study did not assess participants’ urban or rural background, living situation, or socioeconomic status beyond parental education, which may limit the contextual interpretation of the findings, as these factors could independently shape both fermented food accessibility and consumption patterns. Finally, fermented food intake was assessed based on frequency rather than comprehensive dietary patterns, which may not fully capture habitual consumption behaviours. Despite these limitations, the findings provide valuable insights into the challenges of translating nutrition-related awareness into sustainable dietary behaviours. Future studies incorporating more detailed dietary assessment methods, including portion size, microbial content, and product type, would provide a more precise evaluation of fermented food consumption.

## Data Availability

The datasets generated and/or analysed during the current study are not publicly available due [privacy and ethical considerations related to human participant data] but are available from the corresponding author on reasonable request.
